# Young infants’ exposure to parabens: lotion use as a potential source of exposure

**DOI:** 10.1038/s41370-025-00756-4

**Published:** 2025-02-15

**Authors:** Elizabeth Boxer, Yilin Zhong, Jessica Levasseur, Heather M. Stapleton, Kate Hoffman

**Affiliations:** Nicholas School of the Environment, Duke University, Durham, NC USA

**Keywords:** Parabens, Infants, Exposure, urine, personal care products

## Abstract

**Background:**

Parabens are widely used as antimicrobials in personal care products and pharmaceuticals. While previous studies demonstrate paraben exposure is ubiquitous, data investigating infants’ exposure is limited.

**Objective:**

We sought to characterize infants’ exposure to parabens and identify factors associated with higher levels of exposure.

**Methods:**

Families enrolled in the CHildren’s Immune ResPonse Study between 2016-2018. Parents completed questionnaires, providing information on demographics and lifestyle factors. Urine samples were collected when infants were 1 to 3 months old (*n* = 71) and 12 months old (*n* = 29), with 18 infants evaluated at both ages. Parabens were measured in urine samples using liquid chromatography tandem mass spectrometry and served as an indicator of exposure.

**Results:**

Methylparaben (MP), ethylparaben (EP), and propylparaben (PP) were detected in >70% of urine samples, and concentrations ranged several orders of magnitude (specific-gravity-corrected medians: MP = 25.4 PP = 3.55; EP = 0.90 ng/mL). Butylparaben was detected less frequently (<50%). Paraben concentrations were lower than those reported for older children and adults; however, we did not find statistically significant differences in paraben concentrations by infant age. Correlations between measurements taken over time were poor, suggesting paraben exposure is variable, and multiple measurements are needed to capture cumulative exposure information. We observed differences in exposure by race/ethnicity and socioeconomic status; non-White infants and infants whose parents completed less education had higher paraben exposure. Recent lotion usage strongly predicted paraben exposure in 1–3-month-olds. For example, infants using lotion in the past seven days had urinary MP concentrations 355% higher than infants without lotion usage (e^ß^ = 4.55, 95% Confidence Interval = 1.68, 12.55, *p* < 0.001). Together, our results suggest infants are ubiquitously exposed to parabens and personal care product use may be an important source of exposure.

**Impact:**

- To the best of our knowledge, this is the first paper to report paraben levels and evaluate predictors of exposure in infants. This study supports the hypothesis that universal exposure to parabens extends to infants, as indicated by urinary biomarker concentrations. Of the predictors evaluated, lotion use in the last seven days was the strongest predictor of exposure in 1-3-month-olds. Given infant paraben levels are strongly correlated to lotion use, there may be an opportunity for parents to reduce paraben exposure by limiting its application or consulting ingredient labels to ensure no parabens are present.

## Introduction

Parabens are a group of antimicrobial compounds commonly added to a variety of products, including cosmetics, personal care products, food products, and pharmaceuticals to increase their shelf life [1]. Parabens, including butylparaben (BP), ethylparaben (EP), methylparaben (MP), and propylparaben (PP), inhibit the growth of harmful bacteria and mold, thereby protecting products and consumers [1]. However, recent epidemiologic and toxicologic research suggests that elevated exposure to parabens may be related to a number of adverse health outcomes, including disruption of endocrine function, decreased fertility and reproductive harms, adverse birth outcomes, and increased risk of certain cancers [2–6].

Once absorbed, parabens are rapidly metabolized and excreted in the urine, which is commonly used as an indicator of exposure in biomonitoring studies [7, 8]. Their half-life is generally accepted to be less than 24 hours [1, 9]; however, studies of paraben exposure have been conducted among various groups of people and have shown widespread detection of parabens in occupational cohorts and the general population [10–12]. It is well established that the general adult population is continuously and repetitively exposed to parabens through the use of everyday personal care products (PCP) and cosmetics and in smaller amounts from pharmaceuticals, food sources, and the indoor environment [8, 13–16]. Infants are also highly likely to be exposed, as parabens have been found in breastmilk, milk-based infant formula, infant socks, and baby care products such as lotion and sunscreen [17–20]. Prior research suggests exposure varies by demographic characteristics and individual behaviors. For example, adult women have higher urinary concentrations of parabens compared to men, likely due to the fact that women tend to use more PCPs [12, 21]. This is supported by a study of young children aged 3–6 years, whose PCP use varied little by sex, which found this trend in exposure differences based on sex is nonexistent [22]. Racial differences in paraben exposure in female adults have also been observed and are typically attributed to social and cultural patterns of PCP use and/or differential access to products with fewer chemicals [21, 23–25].

While most paraben studies focus on adults and adolescents, little is known about paraben levels and predictors of exposure in infants. Previous data suggests paraben levels increase with age, however infants and young children are uniquely susceptible to environmental contaminants given that they have more skin surface area, breathe more air, drink more water, and eat more food in proportion to their body weight than adults [21]. Additionally, infancy is a unique time of rapidly changing behavioral patterns that can shift sources of exposure, as infants typically begin crawling and teething between 6-10 months and begin a more varied diet as they near one year of age [26]. Evaluating these exposure patterns is critical, as the rapidly growing organ systems of young children are more susceptible to toxic chemicals than adults [27, 28].

The primary objectives of this study were to evaluate exposure to parabens in a cohort of infants (at two time points; 1-to-3-months-old and 12-months-old) and to identify predictors of higher levels of exposure. Identifying characteristics and lifestyle factors contributing to higher levels of exposure may provide an opportunity for interventions, potentially reducing exposure and associated adverse health impacts among children.

## Material and methods

### Study population

Participants were recruited for the CHildren’s Immune ResPonse (CHIRP) Study by team members during routine care appointments at Duke Pediatric Primary Care Clinics in the Durham, North Carolina region between 2016 and 2018 [29]. The study was introduced by the child’s physician and interested families met with the study team for enrollment. The Duke University Health System Institutional Review Board reviewed and approved human subjects’ participation and protocols (Pro00069015). Infants were typically enrolled between 1 and 3 months of age (study visit 1, *n* = 71). At enrollment, each infant provided a single urine sample, and their parents or legal guardians completed a questionnaire with study personnel about the child’s environment, lifestyle, health, and diet. When enrolled children reached 12 months of age, they provided a second urine sample, and their parents completed a second study questionnaire with study staff (*n* = 18). An additional 11 children were recruited for the 12-month visit and provided data and samples at study visit 2 only. The results are expressed separately for the two study visits as we hypothesize exposures and predictors of exposure vary by age.

### Study questionnaire

Study questionnaires were completed by parents when the children were between 1 and 3 months old (study visit 1) and at 12 months of age (study visit 2). Questionnaires were administered by trained staff, who documented answers, and included demographic information (e.g., race and ethnicity, family income, parental education) and information regarding children’s home environments, such as the year of construction and the type of home. Information about the child’s routine and habits was also collected, including whether a rash cream and/or lotion had been applied to the child’s skin in the previous seven days and whether the child attended daycare. In addition, detailed data was collected regarding the child’s diet from birth through age 12 months, including information on breastfeeding, formula feeding, and the introduction of solid foods. Outdoor temperature (daily average) for the day samples were collected was recorded by the study team using data from the National Weather Service.

### Urine collection and analysis

Standard pediatric urine collection bags (Medline MDS190510, Northfield, Illinois, U.S.A.) were used to collect urine samples [30]. Extraction and analysis of small pieces (4 cm^2^) of the bags confirmed all parabens were below detection limits and as a result, bags were unlikely to have contaminated samples (results not shown). Urine samples were primarily collected during the study visits in the clinic; however, parents were provided several bags to collect a sample at home if a child failed to urinate while at the clinic. Each sample was required to have at least 5 mL of urine and multiple voids were combined, if necessary. After each void, bags were emptied into a polyethene jar and stored at −20 °C while awaiting analysis. If a sample was collected at home, it was stored on ice and picked up by study staff the same day.

Prior to extraction, specific gravity (SG) was measured in each sample using a digital handheld refractometer (Atago, Bellevue, WA, U.S.A.). Samples were then analyzed for MP, PP, BP and EP based on an adapted protocol that was detailed previously [31]. Briefly, urine samples (1 mL) were incubated overnight for approximately 12 hours at 37 °C with 1.75 mL of 1 M sodium acetate buffer solution (pH 5),100 µL of a deconjugation enzyme solution (final concentration 1000 units/mL β-glucoronidase and 33 units/mL sulfatase activity) and 50 µL of each internal standard (_13_C-methyl, -ethyl, -propyl and -butyl parabens, present as a mixed stock). After incubation, solid phase extraction was conducted to isolate the parabens from urine using an Empore C18SD column. Parabens were eluted using 8 mL of methanol and the final extract was concentrated under purified nitrogen. Final extracts were each spiked with 50 ng of recovery standard (D_4_-methyl- and propylparaben, present as a mixed stock) then filtered through a 25 mm 0.2 μm PTFE membrane filter before analysis by liquid chromatography tandem mass spectrometry (LC-MS/MS).

LC-MS/MS parameters were previously reported [31]. Mobile phases consisted of 0.8 mM ammonium acetate in (A) methanol and (B) LCMS water, and the flow rate was 0.4 mL/min. Initial conditions were 70% B held for 3 min, following a gradient to 95% A by 4 min and then increased to 98% A by 9 min. This ratio was held until 12.5 minutes before ramping down to 70% B from minutes 13 to 18. Further analysis parameter details can be found in Table S1.

Laboratory processing blanks (*n* = 8) and a urine reference material (*n* = 3; SRM 3673; NIST) were analyzed with samples for quality assurance and quality control. Raw data for urinary paraben concentrations were blank subtracted (using average levels in laboratory processing blanks) before analysis to obtain the paraben concentration in the urine. Method detection limits (MDLs) were defined as three times the standard deviation of lab processing blank concentrations for each paraben. Recovery of the internal standards across all samples and recovery of parabens within the standard reference material are reported in Tables S2 and S3, respectively.

Prior to statistical analysis, paraben concentrations were adjusted to account for urine dilution using SG as we hypothesize that dilution impacts paraben concentrations in urine. As young infants have a liquid diet and their kidneys do not concentrate urine efficiently, their urine is usually highly diluted. SG was corrected for using existing methods [32].

### Statistical analysis

All statistical analyses were performed using R version 4.4.1. Statistical analysis began with a broad evaluation of the collected data and data distributions. We determined detection frequencies for each paraben. When a paraben was detected in >70% of samples overall, we imputed values below the MDL as MDL/2, prior to correction for specific gravity [33] and the compounds were analyzed continuously. Based on Shapiro-Wilk’s normality tests, we found that the concentrations of all parabens were not normally distributed in urine (*p* < 0.001) and distributions were highly skewed. Thus, we used non-parametric statistics or natural log-transformed biomarker concentrations to improve normality in further statistical analysis. For compounds detected in <70% of samples, we conducted analyses dichotomized by detection (detected or not detected in each sample).

We examined correlations between frequently detected parabens in urine at both early (~1–3 months of age) and late infancy (~12 months of age). Among infants with data at both time points, we examined the correlation between early and late infancy paraben metabolite levels using Spearman correlations. We also examined if there were variations in metabolite concentration levels between time points using a paired Wilcoxon rank-sign test. For all categorical potential exposure predictors, we performed a chi-square test of independence to evaluate differences in the covariate distributions by age (i.e., 1–3 months old vs. 12 months old).

Furthermore, we analyzed relationships between potential predictors of exposure and natural log-transformed biomarker concentrations (compounds detected in >70% of samples) using adjusted and unadjusted linear regression models. In the adjusted model, we accounted for race/ethnicity and parents’ highest educational attainment, which were selected based on hypothesized relationships with paraben exposures and other potential predictor variables of interest. Additionally, we assessed variance inflation factors (VIFs) to confirm that multicollinearity among the independent variables did not impact the results [34]. Using a similar approach, we also conducted logistic regression predicting the odds of detection for less frequently detected biomarkers.

These analyses were stratified by age because we hypothesized a priori that exposure differs by age, especially as behaviors change rapidly from early to late infancy. Accordingly, analyses sought to provide insights into changes in factors contributing to paraben exposure over the first year of life. Exposure predictors evaluated in this study include demographic characteristics, such as children’s race/ethnicity (non-Hispanic White vs. another race or ethnicity), parents’ highest educational attainment (college degree or less vs. graduate degree), parents’ household income level (<$80,000 vs. $80,000+) and the sex of the child. We also evaluated potential dietary exposures by examining breastfeeding status. For these analyses, we combined those who previously breastfed but were not currently breastfed and those who never breastfed into one category due to the low sample size of these two categories (i.e., current (1–3 month old *n* = 59, 12 month old *n* = 16) vs. never or prior (1–3 month old *n* = 12, 12 month old *n* = 12). In addition, we evaluated whether there was an association between paraben concentrations and the use of personal care products, such as lotion and rash cream (questionnaires asked whether the product was used in the past 7 days or not). We also examined whether the infant attended daycare. Outdoor high temperature on the day of urine collection was also considered in our analysis as an exposure predictor, as previous studies have shown that many temperature-related factors are likely to influence exposure [22]. It is unclear what may be driving this trend however, we hypothesize that individual behavior may change with outdoor temperature. For example, in winter, when the air in North Carolina tends to be drier, we anticipated higher rates of lotion usage.

Beta coefficients from regression models were exponentiated for easier interpretation. For categorical variables, exponentiated coefficients provide multiplicative changes in urinary paraben concentrations relative to the reference category.

## Results

### Demographic and lifestyle characteristics

Of the total cohort of 83 infants, 72 provided a urine sample and questionnaire at 1–3-months of age, 18 provided a questionnaire and urine samples at both time points, and an additional 11 participants provided a urine sample and questionnaire only at 12 months of age. One urine sample was excluded from analysis due to very low SG (≤1.00001), thus, our final sample size was 82 infants who contributed 100 unique urine samples.

Demographic and lifestyle characteristics of study participants are shown in Table 1. Most infants from both study visits were non-Hispanic White (visit 1: 69%; visit 2: 76%) and came from a home where at least one parent had completed a graduate degree (visit 1: 63%; visit 2: 66%). Family income was more split with about half of households from both visit 1 and 2 making more than $80,000/year. Infant sex skewed heavily towards male at both study visits, likely due to the fact that the pediatric urine collection bags are easier to use on male genital anatomy.

**Table 1:** CHIRP study population demographic and lifestyle characteristics (*n* = 82 unique infants with 100 urine samples).

Characteristic	1-3 Months of Age (*n* = 71)	12 Months of Age (*n* = 29)	*p*-value^a^
**Sex**			
Female	25 (35.2%)	6 (20.7%)	0.24
Male	46 (64.8%)	23 (79.3%)	
**Race**			
Non-Hispanic White	49 (69.0%)	22 (75.9%)	0.68
Another Race/Ethnicity^b^	22 (31.0%)	7 (24.1%)	
**Highest educational attainment of parents**			
College degree or less	26 (36.6%)	8 (27.6%)	0.62
Graduate degree	45 (63.4%)	19 (65.5%)	
Missing	0 (0%)	2 (6.9%)	
**Family income**			
<$80,000	31 (43.7%)	9 (31.0%)	0.46
$80,000+	32 (45.1%)	15 (51.7%)	
Missing	8 (11.3%)	5 (17.2%)	
**Breastfeeding status**			
Current	59 (83.1%)	16 (55.2%)	0.01
Never or prior	12 (16.9%)	12 (41.4%)	
Missing	0 (0%)	1 (3.4%)	
**Lotion usage in the past 7 days**			
No	34 (47.9%)	7 (24.1%)	0.06
Yes	37 (52.1%)	21 (72.4%)	
Missing	0 (0%)	1 (3.4%)	
**Rash cream usage in the past 7 days**			
No	32 (45.1%)	14 (48.3%)	0.82
Yes	39 (54.9%)	14 (48.3%)	
Missing	0 (0%)	1 (3.4%)	
**Attending daycare**			
No	54 (76.1%)	20 (69.0%)	0.63
Yes	17 (23.9%)	9 (31.0%)	

^a^*P*-values indicate a difference in a specific characteristic by age as determined by the chi-squared test of independence. ^b^Another Race/Ethnicity includes *n* = 12 non-Hispanic Black, *n* = 2 multi-racial Hispanic, *n* = 1 multi-racial non-Hispanic, *n* = 1 other Hispanic, and *n* = 1 Hispanic White child at 1-3 months of age; *n* = 2 non-Hispanic Black, *n* = 1 multi-racial Hispanic, *n* = 1 multi-racial non-Hispanic, *n* = 1 other non-Hispanic, *n* = 1 non-Hispanic preferred not to answer race, *n* = 1 Hispanic White child at 12 months of age.

At visit 1, 83% of infants were currently breastfeeding, with a drop to 55% at visit 2. The chi-squared test of independence shows that breastfeeding is significantly more common among children at age 1–3 months than at 12 months of age (*p* = 0.01). Lotion use also differed by visit, with 51% of infants at visit 1 having lotion applied in the last seven days compared to 72% at visit 2 (*p* = 0.06). No other characteristics evaluated differed at or near significance between visits.

### Parabens in urine

Detection frequencies and specific gravity-corrected distributions of parabens are displayed in Table 2. Non-specific gravity-corrected paraben distributions are available in the supplemental information (Table S4). Three of the four parabens had a detection frequency >70% in both visits 1 and 2 (MP, EP, PP). The detection frequency of all parabens increased for visit 2 compared to visit 1; however, median concentrations remained relatively similar. MP was measured at the highest median concentration at both time points, followed by PP. Due to the low detection frequency (<70%) of BP, further statistical analyses were conducted as detected vs. non-detected.

**Table 2:** Descriptive summary of specific gravity-corrected paraben concentrations (ng/mL) in 100 urine samples collected from 82 infants in the CHIRP study.

Parabens	Detection frequency (%)	25th percentile	Median	75th percentile	95th percentile
Visit 1: 1–3-Month-Old Infants (*n* = 71)					
Methylparaben	74.6	6.62	27.7	183	6783
Ethylparaben	80.3	0.39	0.80	3.32	28.5
Propylparaben	98.6	0.84	3.62	21.0	284
Butylparaben	40.9	0.02	0.04	0.14	4.31
Visit 2: 12-Month-Old Infants (*n* = 29)					
Methylparaben	93.1	7.77	20.0	39.7	202
Ethylparaben	100.0	0.54	0.90	3.59	13.0
Propylparaben	100.0	0.62	2.94	6.33	41.0
Butylparaben	55.2	0.01	0.03	0.15	1.17

Method detection limits for MP = 0.88, EP = 0.03, PP = 0.01, BP = 0.01 ng/mL.

Spearman correlation coefficients between parabens and between the two visit time points are shown in Table 3. Among 1-3-month-olds, all parabens were significantly correlated with one another. At the 12 month time point, only MP and PP were statistically significantly correlated, although the general pattern of association was the same at both time points. MP and PP had the highest correlations, with r_s_ = 0.79 at 1-3 months age (*p* < 0.001) and r_s_ = 0.83 at 12 months age (*p* < 0.001). This finding is consistent with previous studies demonstrating that MP and PP are highly correlated in biomonitoring studies, likely due to the combined use of both chemicals in pharmaceuticals and personal care products [12, 20, 35]. There was no correlation between the same parabens across the two time points suggesting paraben exposure varies over time, which is to be expected given the short half-life of parabens in the body [9]. We also saw no significant difference between the distribution of paired paraben concentrations measured at 1–3-month-olds and 12-month-olds, and additionally saw no statistically significant difference in paraben concentrations between timepoints after running a Wilcox signed-rank test for the paired data (Fig. S1).

**Table 3:** Spearman correlation coefficients between parabens stratified by age.

		1–3-Month-Old Infants			12-Month-Old Infants		
		MP	EP	PP	MP	EP	PP
**1–3-Month-Old Infants**	MP	1.00					
	EP	0.46*	1.00				
	PP	0.79*	0.40*	1.00			
**12-Month-Old Infants**	MP	0.03			1.00		
	EP		0.32		0.27	1.00	
	PP			0.27	0.83*	0.26	1.00

^*^P < 0.05.

Visit 1: *n* = 71; Visit 2: *n* = 29; Both visits: *n* = 18.

### Predictors of exposure

Urinary biomarkers of MP, EP, and PP were compared to demographic characteristics (sex, race, education, income), lifestyle characteristics (lotion and rash cream usage, breastfeeding status, and daycare attendance), and ambient temperature using unadjusted and adjusted linear regression models.

**Fig. 1:**
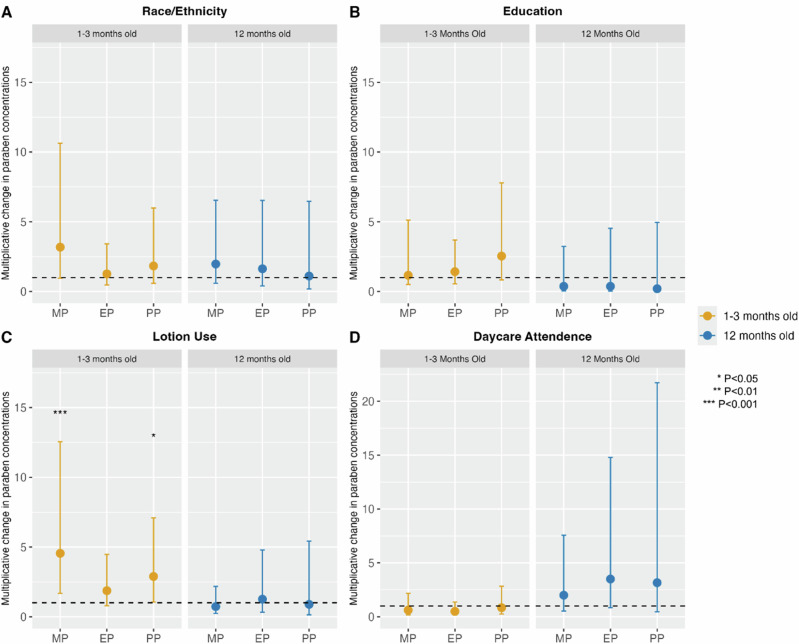
Multiplicative change in infants’ methylparaben (MP), ethylparaben (EP), and propylparaben (PB) urinary biomarkers based on demographic and lifestyle characteristics and 95% confidence intervals from a linear model (all models include race/ethnicity and parental education). Analyses stratified by visit (Visit 1: *n* = 71; Visit 2: *n* = 29). **A** non-White infants (Visit 1: *n* = 22; Visit 2: *n* = 7) compared to non-Hispanic White infants (Visit 1: *n* = 49; Visit 2: *n* = 22). **B** Infants who’s most educated parent had a college degree or less (Visit 1: *n* = 26; Visit 2: *n* = 8) compared to those with a graduate degree (Visit 1: *n* = 45; Visit 2: *n* = 19). **C** Infants who had lotion applied in the last seven days (Visit 1: *n* = 37; Visit 2: *n* = 21) compared to no lotion use in the last seven day (Visit 1: *n* = 34, Visit 2: *n* = 7). **D** Infants who attend daycare (Visit 1: *n* = 37; Visit 2: *n* = 21) compared to infants who do not attend daycare (Visit 1: *n* = 34, Visit 2: *n* = 7).

Among children aged 1-3-months-old, race was a significant predictor of MP exposure after adjusting for parental educational attainment (Fig. 1A; Table S5; unadjusted model in Table S6). MP concentrations were 218% higher in participants who were another race or ethnicity at 1-3 months compared to non-Hispanic White participants (adjusted e^ß^ = 3.18, 95% CI = 0.95 – 10.63, *p* = 0.06). At the 12-month time point, all parabens trended towards higher concentrations in infants of another race or ethnicity, with MP concentrations 97% higher than non-Hispanic White 12-month-olds (adjusted e^ß^ = 1.97, 95% CI = 0.59 – 6.54, *p* = 0.26). In 1-3-month-olds, PP concentrations were 154% higher and MP 61% higher in those whose parents had a college degree or less compared to those with a graduate degree (Fig. 1B; Table S5; PP adjusted e^ß^ = 2.54, 95% CI = 0.83–7.79, *p* = 0.10; MP adjusted e^ß^ = 1.61, 95% CI = 0.50–5.12, *p* = 0.42). Though not statistically significant, all parabens in 12-month-olds showed a trend of lower paraben biomarkers in infants whose most educated parent had a college degree or less in adjusted models (Fig. 1B; Table S5; unadjusted models in Table S6). Associations between other demographic characteristics, such as infant sex and household income, and paraben biomarkers were largely null (i.e., generally close to the reference value and imprecisely estimated) in both adjusted and unadjusted models (Tables S5,S6).

Associations of the urinary biomarkers of MP, EP, and PP were also compared to lotion use, rash cream use, breastfeeding status, and daycare attendance. Lotion use in the last seven days was positively associated with paraben urinary biomarker concentrations. As shown in Fig. 1C, after adjusting for race/ethnicity and parental education, 1–3-month-olds who had lotion applied in the last 7 days had 87% higher levels of EP (e^ß^ = 1.87, 95% CI = 0.79–4.47, *p* = 0.15), 189% higher levels of PP (e^ß^ = 2.89, 95% CI = 1.06–7.90, *p* = 0.04), 355% higher concentrations of MP (e^ß^ = 4.55, 95% CI = 1.68–12.55, *p* < 0.001) than infants who did not have lotion applied (adjusted associations Table S7 and unadjusted associations Table S8) As lotion use appeared to vary by race/ethnicity, a Chi-squared test with Yates’ continuity correction was conducted, and no significant differences between the frequency of lotion use between White and non-White participants at both visits were observed (Table S9). In other analyses, the impact of breastfeeding, rash cream use, and daycare attendance was generally minimal on paraben concentrations and associations were not statistically significant, though 12-month-olds who attended daycare trended towards having increased paraben concentrations (Tables S7, S8). Outdoor temperature was also evaluated and was not found to be a significant predictor of paraben exposure (Table S10).

Additionally, estimated odds of BP urinary biomarker detection based on our predictors of exposure was assessed (Tables S11,S12). These results were generally non-significant, though in our adjusted analysis 12-month-old females were more likely to have butylparaben detection than males (OR = 3.43, 95% CI = 0.40 – 29.49, *p* = 0.26). Additionally at age 1–3 months, in both the adjusted and unadjusted analysis, BP was more likely to be detected in warmer temperatures (Table S13; adjusted OR = 1.09, 95% CI = 1.02 – 1.18, *p* = 0.02). These associations were reduced and not statistically significant in the 12-month-olds.

## Discussion

To the best of our knowledge, this is the first paper to report paraben levels and evaluate predictors of paraben exposure in infants. Of the predictors evaluated, lotion use in the last seven days was the strongest predictor of urinary paraben biomarkers of exposure in 1-3-month-olds, with lotion users having 189% higher concentrations of PP and 355% higher concentrations of MP compared to infants who did not have lotion applied in the last seven days. Given the correlation between MP, EP, and PP at the 1-3-month visit and their frequent co-use in personal care products, it is not surprising to see these compounds associated with lotion use [15, 20, 31, 36]. These results did not fully translate to the 12-month-olds, though it is worth noting the non-statistically significant positive trend between lotion use and EP holds true, indicating lotion use may still be a source of exposure even in older infants. The lack of statistical significance at the 12-month-old visit when there was such a strong association at the 1-3-month-old visit may reflect changing patterns of exposure as infants age, such as a paraben exposure from a more varied diet and exposure to dust and other products as children begin crawling and teething [37–39].

Both race/ethnicity and parental education levels were also associated with infant MP and PP concentrations in 1–3-month-olds, however, we did not observe the same strength of association between parental educational attainment and parabens at 12 months. These results were not statistically significant in the 12-month-old infants, possibly due to the small sample size of non-White participants (*n* = 7) and parents with a college degree or less at this timepoint (*n* = 8). We hypothesize that these demographic characteristics are a proxy for an unmeasured exposure pathway or source. For instance, there may be differences in product use or behavior by race and ethnicity that were not captured in our questionnaire [40–42]. Importantly, although we did not observe statistical differences in lotion usage by race/ethnicity, the proportion of participants reporting lotion usage in the past seven days tended to be lower among non-Hispanic White participants. Similar relationships between educational attainment and race and ethnicity with parabens were found in the TESIE study, which also conducted in Central North Carolina (among children aged 3–6 years) and have been reported for other types of exposures related to personal care products [22].

Racial and ethnic disparities in childhood health outcomes, such as asthma and early onset of puberty, are well-documented [43–46]. Although various social and behavioral factors may contribute to these differences, recent research highlights that unequal exposure to chemicals in PCPs could play a key role in shaping both exposure levels and health disparities in adults [24, 25, 41, 47]. However, less is known about how PCP use shapes childhood and adolescent exposure, especially across race.

Another interesting finding from this study is that 12-month-olds who attended daycare had higher paraben concentrations. This could be due to a variety of things. Hygiene requirements in daycares, including handwashing standards, may explain this pattern. One study in adults found that mean concentrations of parabens in those who wash their hands with hand soap were 158–520% higher than those who did not use soap [48]; however, this has not been investigated in infants and young children.

We did not observe an association between breastfeeding and infant paraben levels in our study, suggesting maternal paraben concentrations are not driving infant levels via diet. However, there may be other potential familial or household sources of exposure that were not captured in this study. For example, passive exposure from parent or household member PCP use may be driving infants’ exposure to parabens.

Paraben concentrations in this cohort were generally lower than those reported for other U.S. cohorts focusing on older age groups. For example, the 2005–2006 National Health and Nutrition Examination Survey (NHANES) found 6–11-year-olds had a mean concentration of 33.5 ng/mL MP and 3.41 ng/mL PP (non-SG corrected), and the TESIE cohort of 3-6-year-old children found a median SG corrected MP concentration of 57 ng/mL, 8.5 ng/mL PP, 1.4 ng/mL EP [16, 21]. One study in Korea investigated urinary paraben levels in newborns within 48 hours after birth and found a median SG corrected MP concentration of 97 ng/mL, 2.9 ng/mL PP, and 4 ng/mL EP, though these levels may better reflect prenatal exposure than infants’ direct exposure [30]. While additional studies have also evaluated adolescent paraben levels, we are unable to make a comparison to our cohort due to the use of different methods to account for urine dilution and a comparison of unadjusted values is not ideal given the inability of the developing kidney to concentrate urine until approximately 1 year of age [49–53].

Since there were no discernible variations in paraben concentrations between infant sexes, it is possible that gender differences in paraben exposure observed in older cohorts stem from distinct personal care product usage rather than excretion or biological factors [21]. However this conclusion should be considered cautiously as our cohort skewed heavily male, likely because pediatric urine collection bags are easier to use on male genital anatomy and we may have been underpowered to detect more subtle differences in paraben concentrations by sex.

This study provides important insight into paraben levels and predictors of paraben exposure in infants and has several strengths including longitudinal sampling and detailed data collection for infants. However, the results should be interpreted in the context of several study limitations. First, our sample population was relatively small and homogenous with the majority of infants being non-Hispanic White and coming from homes with highly educated parents, which limits our ability to generalize our findings to other, more diverse populations. Importantly, due to small sample sizes for some racial and ethnic groups, we categorize participants as non-Hispanic White or another race or ethnicity. This lumping may have obscured important associations for some racial and ethnic groups. The methods used to collect urine also resulted in a male-heavy cohort, with fewer infants at the 12 month time point. Additionally, the time of day of urine collection was not recorded, which may be related to product use patterns and exposure. Similarly, some samples were collected by parents at home, which may have introduced inconsistencies in sample collection procedures. While we do not anticipate that these procedures biased reported associations, investigating sample collection timing and procedures in future studies may provide additional insights. Parents were asked to report about various characteristics of their infants and what products they use, and we did not verify whether parabens were used in the specific products applied. However, we anticipate that this type of misclassification (i.e., assigning potential paraben exposure to all infants using lotion, not just those using paraben-containing lotion) could have resulted in an underestimation of the true impact of paraben-containing lotion use on infants’ levels of exposure. Lastly, information was not collected on parents’ PCP use practices, which may also have an impact on children’s exposure. Future studies should gather more information on more specific product use to validate the presence of parabens, as well as collect product use frequency and product use for the larger household, which may play a role in early childhood paraben exposure.

Taken together, data from the CHIRP cohort study support the hypothesis that universal exposure to parabens extends to infants, as indicated by biomarker concentrations. While paraben levels in infants may be lower than in other age groups, monitoring and classifying their exposure is still of importance as infants may be more susceptible to environmental chemicals [28]. Additionally, their rapid development and underdeveloped defense mechanisms can potentially make these exposures more harmful than later life exposure. Given young infant paraben levels are strongly correlated to lotion use, there may be an opportunity for parents to reduce paraben exposure by limiting its application or consulting ingredient labels to ensure no parabens are present. Future research should prioritize understanding racial/ethnic and socioeconomic disparities in paraben exposure to identify populations at higher risk, address potential inequities in exposure sources (e.g., personal care products marketed to specific groups), and inform targeted interventions that can reduce disproportionate health burdens among vulnerable communities.

## Supplementary information

Supplementary information

## Data Availability

Additional data available from the corresponding author upon request.
