# Supplementary material for: Young infants’ exposure to parabens: lotion use as a potential source of exposure
Source: J Expo Sci Environ Epidemiol. 2025 Feb 15;35(6):1003–10. doi: 10.1038/s41370-025-00756-4 (PMC12583126; doi:10.1038/s41370-025-00756-4)
Supplement: Supplementary file 1 — Supplementary information [file 41370_2025_756_MOESM1_ESM.docx]

**Supplemental Information**

Title: Young infants’ exposure to parabens: lotion use as a potential source of exposure

Authors: Elizabeth Boxer^1†^, Yilin Zhong^1†^, Jessica Levasseur^1^, Heather M. Stapleton^1^, and Kate Hoffman^1*^

Affiliations:

^1^ Nicholas School of the Environment, Duke University, Durham, NC, USA

**^†^ Authors Contributed Equally**

***Corresponding Author**

Kate Hoffman

Duke University

Nicholas School of the Environment

9 Circuit Drive, Box 90328

Durham, NC 27708, USA

Telephone: (919)684-6952

Email: kate.hoffman@duke.edu

**Supplemental Information Contents**

***Table S1:*** *m/z for parabens……………………………………………………………………….…page 3*

***Table S2:*** *Percent recovery of isotopically labelled parabens in urine samples and blanks……………………….…………………………………………………………………………..page 3*

***Table S3:*** *Levels of urinary metabolites measured in lab processing blanks and SRM 3673. ……………………….……………………………………………………………………………….....page 3*

***Table S4:*** *Descriptive summary of non-specific gravity-corrected paraben concentrations (ng/mL) in urine samples collected from infants in the CHIRP study..………………………..page 4*

***Figure S1****: Boxplots of paraben levels in infants’ urine…………….....…………………….....page 5*

***Table S5:*** *Multiplicative change in infants’ paraben urinary biomarker based on demographic characteristics(adjusted)…………………………………………………………………………….page 6*

***Table S6:*** *Multiplicative change in infants’ paraben urinary biomarker based on demographic characteristics(unadjusted)……………………………………………..…………………………..page 7*

***Table S7:*** *Multiplicative change in infants’ paraben urinary biomarker based on lifestyle factors (adjusted)…………………………………………………………………..………………………….page 8*

***Table S8:*** *Multiplicative change in infants’ paraben urinary biomarker based on lifestyle factors (unadjusted)…………………………………………………………………..……………………....page 9*

***Table S9:*** *Lotion use by visit and race/ethnicity……………………………..……………………..page 10*

***Table S10:*** *Multiplicative change in infants’ paraben urinary biomarker concentration for a one-degree Celsius change in outdoor air temperature (adjusted and unadjusted)……....page 11*

***Table S11:*** *Odds ratio (OR) of butylparaben urinary biomarker detection based on demographic and lifestyle characteristics (adjusted). ………………………..……………………………....page 12*

***Table S12:*** *Odds ratio (OR) of butylparaben urinary biomarker detection based on demographic and lifestyle characteristics (unadjusted)…………………..…………………………………..page 13*

***Table S13:*** *Odds ratio (OR) of butylparaben urinary biomarker detection based on demographic characteristics (adjusted and unadjusted) …………..………………………..page 14*

**Table S1:** m/z for parabens.

| **Compound** | **Transition** | **Internal Standard** | **Transition** |
| --- | --- | --- | --- |
| Methylparaben | 150.9/92.0 | ^13^C Methylparaben | 157.1/98.0 |
| Ethylparaben | 165.1/92.0 | ^13^C Ethylparaben | 171.1/98.0 |
| Propylparaben | 179.1/92.0 | ^13^C Propylparaben | 185.1/98.0 |
| Butylparaben | 193.1/92.0 | ^13^C Butylparaben | 199.1/98.0 |

**Table S2:** Percent recovery of 50 ng isotopically labelled parabens as measured in urine samples and blanks.

| **Chemical** | **Average percent recovery of internal standard in all samples** | **Average percent recovery in blanks**  **(n = 8)** |
| --- | --- | --- |
| Methylparaben | 103% | 102% |
| Ethylparaben | 97% | 102% |
| Propylparaben | 109% | 113% |
| Butylparaben | 164% | 131% |

**Table S3:** Levels of urinary metabolites measured in lab processing blanks (n = 8) and SRM 3673 (n = 3).

| **Chemical** | **Average measurement in SRM 3673 (ng/mL)** | **Reference values in SRM 3673 (ng/g)** | **Average percent of reference values** |
| --- | --- | --- | --- |
| Methylparaben | 71.41 | 79.5 | 90 |
| Ethylparaben | 7.10 | 10.3 | 69 |
| Propylparaben | 15.54 | 21.6 | 72 |
| Butylparaben | 0.69 | 1.11 | 62 |

**Table S4:** Descriptive summary of non-specific gravity-corrected paraben concentrations (ng/mL) in 100 urine samples collected from 82 infants in the CHIRP study. Method detection limits for MP=0.88, EP=0.03, PP=0.01, BP=0.01 ng/mL.

| Parabens | Detection Frequency (%) | 25th Percentile | Median | 75th Percentile | 95th Percentile |
| --- | --- | --- | --- | --- | --- |
| Visit 1: 1–3-Month-Old Infants  (n = 71) |  |  |  |  |  |
| Methylparaben | 74.6 | 0.67 | 3.41 | 26.8 | 494.9 |
| Ethylparaben | 80.3 | 0.05 | 0.13 | 0.38 | 3.58 |
| Propylparaben | 98.6 | 0.10 | 0.44 | 2.76 | 30.3 |
| Butylparaben | 40.9 | -- | -- | 0.02 | 0.81 |
| Visit 2: 12-Month-Old Infants (n = 29) |  |  |  |  |  |
| Methylparaben | 93.1 | 2.78 | 7.74 | 18.5 | 91.4 |
| Ethylparaben | 100.0 | 0.10 | 0.42 | 1.38 | 4.78 |
| Propylparaben | 100.0 | 0.14 | 1.14 | 3.54 | 25.1 |
| Butylparaben | 55.2 | -- | -- | 0.07 | 0.67 |

**Figure S1:** a. Boxplot of ln transformed paraben levels stratified by visit collection. Visit 1 (1-3 months old): n = 71; Visit 2 (12 months old): n = 29. b. Boxplot of ln transformed paraben levels restricted to individuals with samples at both 1-3 months old and 12 months old stratified by sample visit: n = 18. P-values are derived from a Wilcox signed-rank test accounting for paired data.


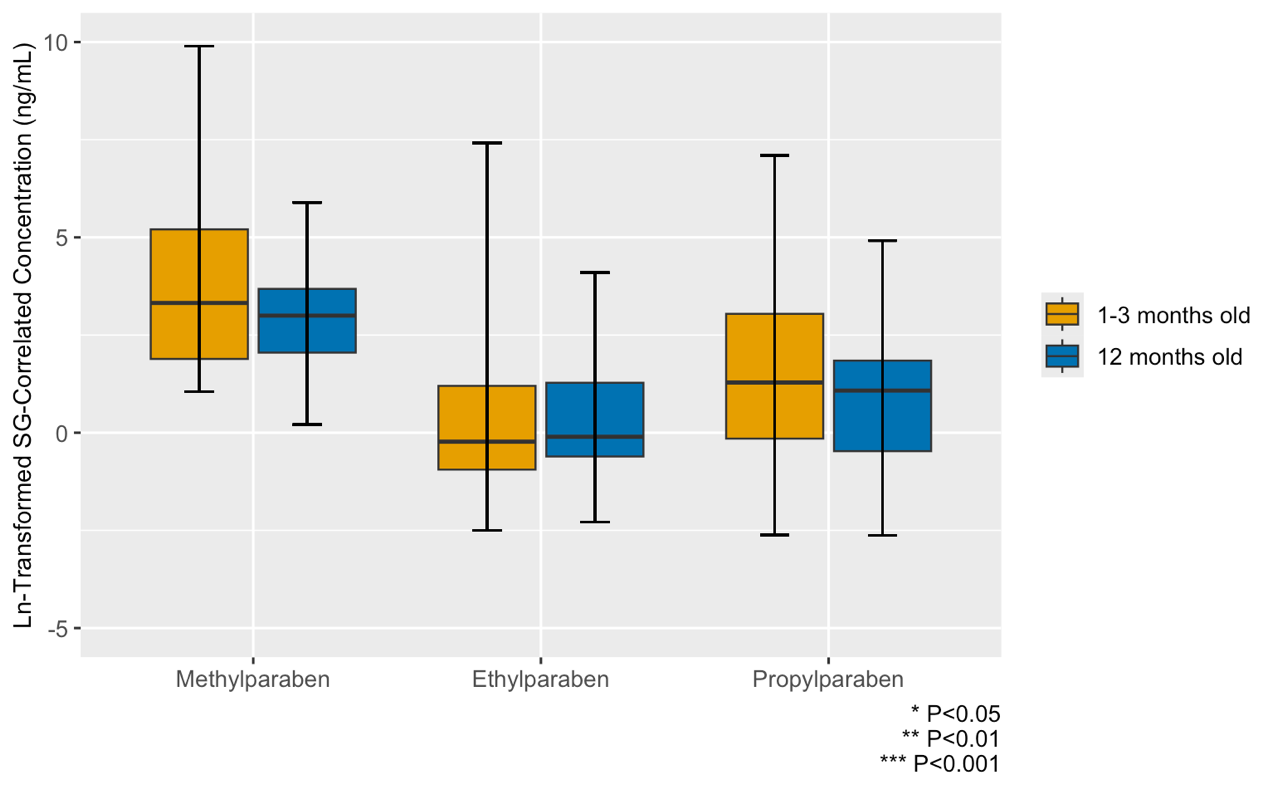


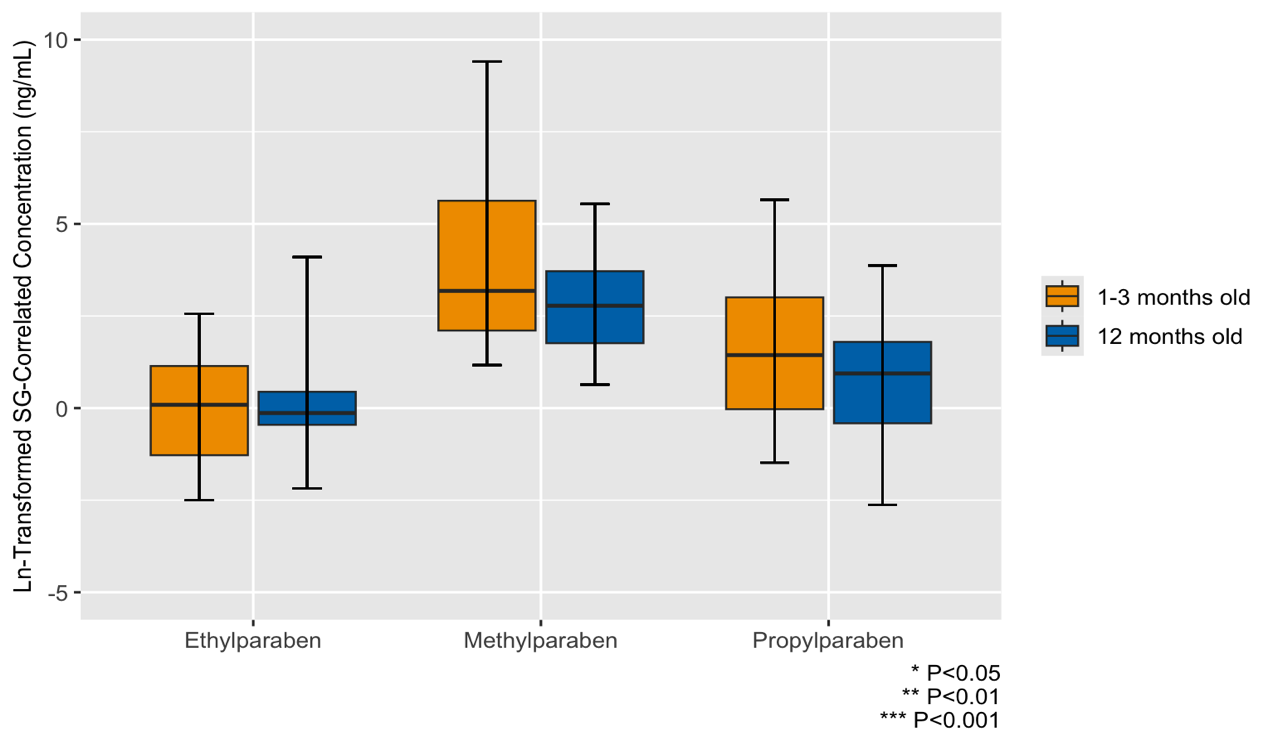


**Table S5:** Estimated multiplicative change in infants’ paraben urinary biomarker based on demographic characteristics compared to a reference group, stratified by visit (1-3-month-olds and 12-month-olds) and 95% confidence intervals based on a linear model adjusted for race/ethnicity and for parents’ highest educational attainment. Visit 1: n = 71; Visit 2: n = 29.

|  |  | **Methylparaben** | | | | | | **Ethylparaben** | | | | | | **Propylparaben** | | | | | |
| --- | --- | --- | --- | --- | --- | --- | --- | --- | --- | --- | --- | --- | --- | --- | --- | --- | --- | --- | --- |
|  |  | **Age 1-3 Months** | | | **Age 12 Months** | | | **Age 1-3 Months** | | | **Age 12 Months** | | | **Age 1-3 Months** | | | **Age 12 Months** | | |
| **Demographic characteristic** |  | e^ß^ | 95% CI | P-Value | e^ß^ | 95% CI | P-Value | e^ß^ | 95% CI | P-Value | e^ß^ | 95% CI | P-Value | e^ß^ | 95% CI | P-Value | e^ß^ | 95% CI | P-Value |
| **Ethnicity** |  |  |  |  |  |  |  |  |  |  |  |  |  |  |  |  |  |  |  |
|  | Another race/ ethnicity | 3.18 | 0.95, 10.63 | **0.06** | 1.97 | 0.59, 6.54 | 0.26 | 1.26 | 0.47, 3.42 | 0.69 | 1.63 | 0.40, 6.52 | 0.47 | 1.83 | 0.58, 5.99 | 0.29 | 1.11 | 0.19, 6.47 | 0.90 |
|  | Non-Hispanic White | Reference | | | Reference | | | Reference | | | Reference | | | Reference | | | Reference | | |
| **Educational Attainment** |  |  |  |  |  |  |  |  |  |  |  |  |  |  |  |  |  |  |  |
|  | College degree or less | 1.61 | 0.50, 5.12 | 0.42 | 0.37 | 0.04, 3.23 | 0.21 | 1.42 | 0.55, 3.69 | 0.46 | 0.37 | 0.03, 4.53 | 0.42 | 2.54 | 0.83, 7.79 | 0.10 | 0.20 | 0.01, 4.95 | 0.31 |
|  | Graduate degree | Reference | | | Reference | | | Reference | | | Reference | | | Reference | | | Reference | | |
| **Income** |  |  |  |  |  |  |  |  |  |  |  |  |  |  |  |  |  |  |  |
|  | Low (<$80,000) | 0.85 | 0.21, 3.43 | 0.82 | 1.57 | 0.40, 6.13 | 0.50 | 1.42 | 0.54, 3.73 | 0.46 | 0.24 | 0.06, 1.04 | 0.06 | 0.86 | 0.22, 3.28 | 0.82 | 1.82 | 0.25, 13.32 | 0.54 |
|  | High (>$80,000) | Reference | | | Reference | | | Reference | | | Reference | | | Reference | | | Reference | | |
| **Sex** |  |  |  |  |  |  |  |  |  |  |  |  |  |  |  |  |  |  |  |
|  | Female | 0.99 | 0.33, 2.96 | 0.99 | 0.91 | 0.24, 3.51 | 0.89 | 0.87 | 0.35, 2.14 | 0.75 | 2.14 | 0.48, 9.48 | 0.30 | 1.19 | 0.41, 3.41 | 0.75 | 0.53 | 0.08, 3.77 | 0.51 |
|  | Male | Reference | | | Reference | | | Reference | | | Reference | | | Reference | | | Reference | | |

**Table S6:** Estimated multiplicative change in infants’ paraben urinary biomarker based on demographic characteristics compared to a reference group, stratified by visit (1-3-month-olds and 12-month-olds) and 95% confidence intervals based on an unadjusted linear model. Visit 1: n = 71; Visit 2: n = 29.

|  |  | **Methylparaben** | | | | | | **Ethylparaben** | | | | | | **Propylparaben** | | | | | |
| --- | --- | --- | --- | --- | --- | --- | --- | --- | --- | --- | --- | --- | --- | --- | --- | --- | --- | --- | --- |
|  |  | **Age 1-3 Months** | | | **Age 12 Months** | | | **Age 1-3 Months** | | | **Age 12 Months** | | | **Age 1-3 Months** | | | **Age 12 Months** | | |
| **Demographic characteristic** |  | e^ß^ | 95% CI | P-Value | e^ß^ | 95% CI | P-Value | e^ß^ | 95% CI | P-Value | e^ß^ | 95% CI | P-Value | e^ß^ | 95% CI | P-Value | e^ß^ | 95% CI | P-Value |
| **Ethnicity** |  |  |  |  |  |  |  |  |  |  |  |  |  |  |  |  |  |  |  |
|  | Another race/ ethnicity | 3.83 | 1.25, 11.69 | **0.02** | 2.24 | 0.69, 7.23 | 0.17 | 1.45 | 0.58, 3.64 | 0.42 | 1.67 | 0.44, 6.40 | 0.44 | 2.68 | 0.90, 8.03 | 0.08 | 1.37 | 0.24, 7.74 | 0.71 |
|  | Non-Hispanic White | Reference | | | Reference | | | Reference | | | Reference | | | Reference | | | Reference | | |
| **Educational Attainment** |  |  |  |  |  |  |  |  |  |  |  |  |  |  |  |  |  |  |  |
|  | College degree or less | 2.44 | 0.82, 7.29 | 0.11 | 1.35 | 0.42, 4.36 | 0.60 | 1.55 | 0.64, 3.73 | 0.33 | 0.53 | 0.14, 1.99 | 0.33 | 3.18 | 1.12, 8.99 | **0.03** | 1.61 | 0.30, 8.62 | 0.56 |
|  | Graduate degree | Reference | | | Reference | | | Reference | | | Reference | | | Reference | | | Reference | | |
| **Income** |  |  |  |  |  |  |  |  |  |  |  |  |  |  |  |  |  |  |  |
|  | Low (<$80,000) | 1.52 | 0.49, 4.72 | 0.46 | 2.01 | 0.61, 6.61 | 0.24 | 1.05 | 0.48, 2.29 | 0.90 | 0.35 | 0.09, 1.30 | 0.11 | 1.56 | 0.52, 4.65 | 0.41 | 2.03 | 0.36, 11.42 | 0.40 |
|  | High (>$80,000) | Reference | | | Reference | | | Reference | | | Reference | | | Reference | | | Reference | | |
| **Sex** |  |  |  |  |  |  |  |  |  |  |  |  |  |  |  |  |  |  |  |
|  | Female | 1.03 | 0.34, 3.18 | 0.95 | 0.85 | 0.23, 3.05 | 0.79 | 0.88 | 0.36, 2.17 | 0.79 | 2.37 | 0.58, 9.53 | 0.21 | 1.26 | 0.42, 3.72 | 0.68 | 0.43 | 0.07, 2.62 | 0.34 |
|  | Male | Reference | | | Reference | | | Reference | | | Reference | | | Reference | | | Reference | | |

|  |  | **Methylparaben** | | | | | | **Ethylparaben** | | | | | | **Propylparaben** | | | | | |
| --- | --- | --- | --- | --- | --- | --- | --- | --- | --- | --- | --- | --- | --- | --- | --- | --- | --- | --- | --- |
|  |  | **Age 1-3 Months** | | | **Age 12 Months** | | | **Age 1-3 Months** | | | **Age 12 Months** | | | **Age 1-3 Months** | | | **Age 12 Months** | | |
| **Lifestyle factors** |  | e^ß^ | 95% CI | P-Value | e^ß^ | 95% CI | P-Value | e^ß^ | 95% CI | P-Value | e^ß^ | 95% CI | P-Value | e^ß^ | 95% CI | P-Value | e^ß^ | 95% CI | P-Value |
| **Lotion Use** |  |  |  |  |  |  |  |  |  |  |  |  |  |  |  |  |  |  |  |
|  | Yes | 4.55 | 1.68, 12.55 | **0.00** | 0.73 | 0.24, 2.18 | 0.55 | 1.87 | 0.79, 4.47 | 0.15 | 1.26 | 0.33, 4.79 | 0.72 | 2.89 | 1.06, 7.90 | **0.04** | 0.89 | 0.14, 5.43 | 0.89 |
|  | No | Reference | | | Reference | | | Reference | | | Reference | | | Reference | | | Reference | | |
| **Rash Cream Use** |  |  |  |  |  |  |  |  |  |  |  |  |  |  |  |  |  |  |  |
|  | Yes | 1.66 | 0.53, 5.20 | 0.38 | 0.88 | 0.32, 2.39 | 0.79 | 0.92 | 0.36, 2.39 | 0.87 | 1.22 | 0.38, 3.90 | 0.72 | 0.93 | 0.30, 2.83 | 0.89 | 1.10 | 0.21, 5.69 | 0.90 |
|  | No | Reference | | | Reference | | | Reference | | | Reference | | | Reference | | | Reference | | |
| **Breastfeeding Status** |  |  |  |  |  |  |  |  |  |  |  |  |  |  |  |  |  |  |  |
|  | Currently breastfeeding | 0.93 | 0.20, 4.42 | 0.93 | 1.72 | 0.60, 4.91 | 0.29 | 2.12 | 0.59, 7.55 | 0.24 | 0.45 | 0.14, 1.50 | 0.18 | 1.24 | 0.27, 5.61 | 0.78 | 1.59 | 0.28, 9.11 | 0.58 |
|  | Not currently breastfeeding | Reference | | | Reference | | | Reference | | | Reference | | | Reference | | | Reference | | |
| **Daycare Attendance** |  |  |  |  |  |  |  |  |  |  |  |  |  |  |  |  |  |  |  |
|  | Yes | 0.62 | 0.18, 2.18 | 0.46 | 2.00 | 0.53, 7.57 | 0.29 | 0.50 | 0.18, 1.37 | 0.17 | 3.50 | 0.83, 14.79 | 0.08 | 0.84 | 0.25, 2.83 | 0.77 | 3.16 | 0.46, 21.72 | 0.23 |
|  | No | Reference | | | Reference | | | Reference | | | Reference | | | Reference | | | Reference | | |

**Table S7:** Estimated multiplicative change in infants’ paraben urinary biomarker based on lifestyle factors compared to a reference group, stratified by visit (1-3-month-olds and 12-month-olds) and 95% confidence intervals based on a linear model adjusted for race/ethnicity and for parents’ highest educational attainment. Visit 1: n = 71; Visit 2: n = 29.

**Table S8:** Estimated multiplicative change in infants’ paraben urinary biomarker based on lifestyle factors compared to a reference group, stratified by visit (1-3-month-olds and 12-month-olds) and 95% confidence intervals based on an unadjusted linear model. Visit 1: n = 71; Visit 2: n = 29.

|  |  | **Methylparaben** | | | | | | **Ethylparaben** | | | | | | **Propylparaben** | | | | | | | |
| --- | --- | --- | --- | --- | --- | --- | --- | --- | --- | --- | --- | --- | --- | --- | --- | --- | --- | --- | --- | --- | --- |
|  |  | **Age 1-3 Months** | | | **Age 12 Months** | | | **Age 1-3 Months** | | | **Age 12 Months** | | | **Age 1-3 Months** | | | | **Age 12 Months** | | | |
| **Lifestyle factors** |  | e^ß^ | 95% CI | P-Value | e^ß^ | 95% CI | P-Value | e^ß^ | 95% CI | P-Value | e^ß^ | 95% CI | P-Value | e^ß^ | 95% CI | P-Value | e^ß^ | | 95% CI | P-Value |  |
| **Lotion Use** |  |  |  |  |  |  |  |  |  |  |  |  |  |  |  |  |  | |  |  |  |
|  | Yes | 5.72 | 2.12, 15.39 | **<0.01** | 0.90 | 0.28, 2.84 | 0.85 | 1.99 | 0.86, 6.62 | 0.10 | 1.62 | 0.43, 5.99 | 0.45 | 3.53 | 1.30, 9.53 | **0.01** | 1.06 | | 0.19, 5.89 | 0.95 |  |
|  | No | Reference | | | Reference | | | Reference | | | Reference | | | Reference | | | | Reference | | | |
| **Rash Cream Use** |  |  |  |  |  |  |  |  |  |  |  |  |  |  |  |  |  | |  |  |  |
|  | Yes | 0.92 | 0.31, 2.70 | 0.95 | 0.74 | 0.28, 1.98 | 0.54 | 0.77 | 0.33, 1.82 | 0.55 | 1.38 | 0.45, 4.33 | 0.56 | 0.56 | 0.20, 1.58 | 0.27 | 0.91 | | 0.21, 4.04 | 0.90 |  |
|  | No | Reference | | | Reference | | | Reference | | | Reference | | | Reference | | | | Reference | | | |
| **Breastfeeding Status** |  |  |  |  |  |  |  |  |  |  |  |  |  |  |  |  |  | |  |  |  |
|  | Currently breastfeeding | 0.49 | 0.12, 2.05 | 0.33 | 1.21 | 0.44, 3.29 | 0.70 | 1.38 | 0.44, 4.30 | 0.57 | 0.61 | 0.19, 1.90 | 0.37 | 0.57 | 0.14, 2.27 | 0.42 | 0.97 | | 0.22, 4.36 | 0.97 |  |
|  | Not currently breastfeeding | Reference | | | Reference | | | Reference | | | Reference | | | Reference | | | | Reference | | | |
| **Daycare Attendance** |  |  |  |  |  |  |  |  |  |  |  |  |  |  |  |  |  | |  |  |  |
|  | Yes | 0.55 | 0.16, 1.94 | 0.35 | 1.91 | 0.64, 5.71 | 0.23 | 0.47 | 0.18, 1.25 | 0.13 | 3.29 | 1.02, 10.51 | **0.04** | 0.66 | 0.20, 2.23 | 0.50 | 2.96 | | 0.63, 13.93 | 0.42 |  |
|  | No | Reference | | | Reference | | | Reference | | | Reference | | | Reference | | | | Reference | | | |

**Table S9:** Lotion use by visit and race/ethnicity.

| Age | Race/Ethnicity | Use Lotion (%) | Don’t use lotion (%) | Total | P-value* |
| --- | --- | --- | --- | --- | --- |
| **1-3 Months** | Non-Hispanic White | 22 (45%) | 27 (55%) | 49 | 0.12 |
|  | Another race/ethnicity | 15 (68%) | 7 (32% | 22 |  |
| **12 Months** | Non-Hispanic White | 16 (73%) | 6 (27%) | 22 | 1.00 |
|  | Another race/ethnicity | 5 (71%) | 2 (29%) | 7 |  |

* P-value obtained from Pearson’s Chi-squared test.

**Table S10**: Estimated multiplicative change in infants’ paraben urinary biomarker concentration for a one-degree Celsius change in outdoor air temperature at sample collection, stratified by visit (1-3-month-olds and 12-month-olds) and 95% confidence intervals based on: a. on a linear model adjusted for race/ethnicity and for parents’ highest educational attainment; b. an unadjusted linear model. Visit 1: n = 71; Visit 2: n = 29.

a.

|  | **Age 1-3 Months** | | | **Age 12 Months** | | |
| --- | --- | --- | --- | --- | --- | --- |
| **Parabens** | **e^ß^** | **95% Confidence Limits** | **P-Value** | **Estimate** | **95% Confidence Limits** | **P-Value** |
| Methylparaben | 1.00 | 0.94, 1.08 | 0.92 | 1.03 | 0.96, 1.11 | 0.37 |
| Ethylparaben | 1.04 | 0.98, 1.10 | 0.18 | 0.99 | 0.91, 1.08 | 0.80 |
| Propylparaben | 1.00 | 0.94, 1.07 | 0.91 | 1.06 | 0.94, 1.20 | 0.32 |

b.

|  | **Age 1-3 Months** | | | **Age 12 Months** | | |
| --- | --- | --- | --- | --- | --- | --- |
| **Parabens** | **e^ß^** | **95% Confidence Limits** | **P-Value** | **Estimate** | **95% Confidence Limits** | **P-Value** |
| Methylparaben | 1.07 | 0.94, 1.09 | 0.63 | 1.03 | 0.96, 1.10 | 0.37 |
| Ethylparaben | 1.04 | 0.98, 1.10 | 0.15 | 1.03 | 0.95, 1.12 | 0.44 |
| Propylparaben | 1.01 | 0.94, 1.08 | 0.71 | 1.05 | 0.94, 1.16 | 0.39 |

|  |  | **Butylparaben** | | | | | | |
| --- | --- | --- | --- | --- | --- | --- | --- | --- |
|  |  | **Age 1-3 Months** | | | | **Age 12 Months** | | |
| **Demographic characteristic** |  | OR | | 95% CI | P-Value | OR | 95% CI | P-Value |
| **Ethnicity** |  |  | |  |  |  |  |  |
|  | Another race/ ethnicity | 1.11 | | 0.36, 3.34 | 0.85 | 0.36 | 0.05, 2.46 | 0.29 |
|  | Non-Hispanic White | Reference | | | | Reference | | |
| **Educational Attainment** |  |  | |  |  |  |  |  |
|  | College degree or less | 1.24 | | 0.42, 3.57 | 0.69 | 1.99 | 0.35, 11.37 | 0.44 |
|  | Graduate degree | Reference | | | | Reference | | |
| **Income** |  |  | |  |  |  |  |  |
|  | Low (<$80,000) | 0.71 | | 0.19, 2.46 | 0.59 | 1.68 | 0.24, 2.17 | 0.60 |
|  | High (>$80,000) | Reference | | | | Reference | | |
| **Sex** |  |  | |  |  |  |  |  |
|  | Female | 1.10 | | 0.41, 2.96 | 0.85 | 3.43 | 0.40, 29.49 | 0.26 |
|  | Male | Reference | | | | Reference | | |
| **Lifestyle factors** |  |  | | | |  | | |
| **Lotion Use** |  |  | | | |  | | |
|  | Yes | 1.04 | 0.39, 1.75 | | 0.94 | 0.13 | 0.01, 1.32 | 0.08 |
|  | No | Reference | | | | Reference | | |
| **Rash Cream Use** |  |  | | | |  | | |
|  | Yes | 0.42 | 0.14, 1.21 | | 0.11 | 0.53 | 0.10, 2.66 |  |
|  | No | Reference | | | | Reference | | |
| **Breastfeeding Status** |  |  | | | |  | | |
|  | Currently breastfeeding | 0.76 | 0.18, 3.12 | | 0.69 | 0.35 | 0.06, 2.06 | 0.24 |
|  | Not currently breastfeeding | Reference | | | | Reference | | |
| **Daycare Attendance** |  |  | | | |  | | |
|  | Yes | 0.49 | 0.14, 1.58 | | 0.25 | 1.72 | 0.25, 11.74 | 0.58 |
|  | No | Reference | | | | Reference | | |

**Table S11:** Estimated odds ratio (OR) of infants’ likelihood of butylparaben urinary biomarker detection based on demographic characteristics compared to a reference group, stratified by visit (1-3-month-olds and 12-month-olds) and 95% confidence intervals based on a model adjusted for parents’ highest educational attainment. Visit 1: n = 71; Visit 2: n = 29.

|  |  | **Butylparaben** | | | | | | | | | |
| --- | --- | --- | --- | --- | --- | --- | --- | --- | --- | --- | --- |
|  |  | **Age 1-3 Months** | | | | **Age 12 Months** | | | | | |
| **Demographic characteristic** |  | OR | 95% CI | P-Value | | OR | 95% CI | | | P-Value | |
| **Ethnicity** |  |  |  |  | |  |  | | |  | |
|  | Another race/ ethnicity | 1.21 | 0.42, 3.34 | 0.71 | | 0.52 | 0.08, 2.91 | | | 0.46 | |
|  | Non-Hispanic White | Reference | | | | Reference | | | | | |
| **Educational Attainment** |  |  |  |  | |  |  | | |  | |
|  | College degree or less | 1.29 | 0.48, 3.43 | 0.61 | | 1.85 | 0.35, 11.23 | | | 0.48 | |
|  | Graduate degree | Reference | | | | Reference | | | | | |
| **Income** |  |  |  |  | |  |  | | |  | |
|  | Low (<$80,000) | 0.71 | 0.26, 1.95 | 0.50 | | 1.09 | 0.21, 5.75 | | | 0.92 | |
|  | High (>$80,000) | Reference | | | | Reference | | | | | |
| **Sex** |  |  |  |  | |  |  | | |  | |
|  | Female | 1.12 | 0.41, 2.99 | 0.82 | | 1.83 | 0.28, 12.07 | | | 0.53 | |
|  | Male | Reference | | | | Reference | | | | | |
| **Lifestyle factors** |  |  | | | |  | | | | | |
| **Lotion Use** |  |  | | | |  | | | | | |
|  | Yes | 1.09 | 0.42, 1.82 | | 0.86 | 0.15 | | 0.02, 1.49 | | | 0.11 |
|  | No | Reference | | | | Reference | | | | | |
| **Rash Cream Use** |  |  | | | |  | | | | | |
|  | Yes | 0.44 | 0.17, 1.14 | | 0.10 | 0.56 | | | 0.12, 2.53 | |  |
|  | No | Reference | | | | Reference | | | | | |
| **Breastfeeding Status** |  |  | | | |  | | | | | |
|  | Currently breastfeeding | 0.68 | 0.19, 2.44 | | 0.55 | 0.26 | | 0.05, 1.33 | | | 0.11 |
|  | Not currently breastfeeding | Reference | | | | Reference | | | | | |
| **Daycare Attendance** |  |  | | | |  | | | | | |
|  | Yes | 0.48 | 0.14, 1.50 | | 0.22 | 2.00 | | | 0.38, 10.31 | | 0.41 |
|  | No | Reference | | | | Reference | | | | | |

**Table S12:** Estimated odds ratio (OR) of infants’ likelihood of butylparaben urinary biomarker detection demographic characteristics compared to a reference group, stratified by visit (1-3-month-olds and 12-month-olds) and 95% confidence intervals based on an unadjusted model. Visit 1: n = 71; Visit 2: n = 29.

**Table S13**: Estimated odds ratio (OR) of infants’ likelihood of butylparaben urinary biomarker detection for a one-degree Celsius change in outdoor air temperature sample collection, stratified by visit (1-3-month-olds and 12-month-olds) and 95% confidence intervals based on: a. An unadjusted generalized linear model; b. A generalized linear model adjusted for race/ethnicity and parents’ highest educational attainment. Visit 1: n = 71; Visit 2: n = 29.

a.

|  | **Age 1-3 Months** | | | **Age 12 Months** | | |
| --- | --- | --- | --- | --- | --- | --- |
| **Paraben** | **OR** | **95% Confidence Limits** | **P-Value** | **Estimate** | **95% Confidence Limits** | **P-Value** |
| Butylparaben | 1.09 | 1.02, 1.17 | **0.02** | 0.96 | 0.86, 1.07 | 0.45 |

b.

|  | **Age 1-3 Months** | | | **Age 12 Months** | | |
| --- | --- | --- | --- | --- | --- | --- |
| **Paraben** | **OR** | **95% Confidence Limits** | **P-Value** | **Estimate** | **95% Confidence Limits** | **P-Value** |
| Butylparaben | 1.09 | 1.02, 1.18 | **0.02** | 0.96 | 0.85, 1.08 | 0.49 |
